# If not us, then who? Ethnographic insights into nurses’ role in redesigning a rural health service to meet changing demands

**DOI:** 10.1186/s12913-025-12397-2

**Published:** 2025-02-21

**Authors:** Helle Krone-Hjertstrøm, Bente Norbye, Birgit Abelsen, Aud Uhlen Obstfelder

**Affiliations:** 1 Department of Health and Care Sciences, Faculty of Health Sciences, UiT The Arctic University of Norway, Tromsø, Norway; 2 Telemark Research Institute, Bø, Norway; 3 The Norwegian Centre for Rural Medicine, The Department of Community Medicine, UiT The Arctic University of Norway, Tromsø, Norway; 4 Department of Health Sciences in Gjøvik, Faculty of Medicine and Health Sciences, NTNU, Norwegian University of Science and Technology, Gjøvik, Norway

**Keywords:** Rural healthcare, Service redesign, Municipal inpatient acute care service, Nursing, Nursing work, Primary healthcare research, Qualitative research, Social organisation of healthcare work

## Abstract

**Background:**

The increasing prevalence of chronic diseases and an ageing population challenge healthcare delivery, particularly in rural areas. Redesigning health services to meet these changing demands is crucial, esially in rural healthcare where service providers face capacity and expertise limitations. This paper explores how the rural context influences nurses' roles in the clinic and the process of redesigning a rural emergency clinic in Northern Norway to include a Municipal Inpatient Acute Care Service (MipAC).

**Methods:**

The study combines observations (250 h), several in situ interviews with healthcare personnel, and individual in-depth interviews with nurses (*n* = 8) at the emergency clinic in a rural area in northern Norway. To explore nurses’ role in the clinic and in the redesign process, we were inspired by *the theory of social organisation of healthcare work.*

**Results:**

The study highlights three prominent aspects of the rural dimension that are crucial for understanding the nurses’ role in the redesign process. First, ‘Absent present PCPs’ highlights how nurses ensure continuity of care during the intermittent absence of primary care physicians (PCPs). Second, ‘Learning without doing’ shows how the nurses utilized their competence, past experiences, and understanding of local conditions to adapt standardised guidelines, develop new work processes, and establish a framework that compensates for the lack of practical experience, enabling the clinic to function effecively under potential challanging circumstances. Third, ‘The local nurse' emphasises how the nurses’ connection to the local community influences how they organize care; they are commited to local adaptation and ensuring that "their" residents have acess to what they consider high-quality services.

**Conclusion:**

The nurses’, experience, dedication, and orientation towards the local community were crucial for the redesign process. The nurses ensured the continuiety in the clinic, despite the PCP's sporadic presence, adapted the standardized guidelines to a rural context and were crucial in organising patient care in the rural area. The study shows that the nurses organizing work, which often remains invisible, is essential in the redesign process. The study indicates that the scope of practice for nurses is more extensive in rural areas and should receive greater attention. in both research, practice and health policy

## Introduction

The delivery of healthcare is challenged by the increased prevalence of chronic diseases and an ageing population, particularly in hospital-based care [1]. It is necessary to reduce the pressure on hospitals and establish service delivery models that involve the collaboration of different health professionals and institutions at various administrative levels. Many countries have developed primary healthcare models that offer alternatives to hospitalisation, such as intermediate care units and community hospitals [2–4]. There is a tendency to redesign services and implement changes within the existing service structure [5]. Redesigning health services presents a significant challenge, particularly in rural areas where service providers find that the services they are expected to deliver exceed both their capacity and expertise [6, 7].

Bourke [8] highlights how health services and interventions are developed and shaped by the broader health system, government and social structures and how they are (re) shaped and produced locally to meet the premisses and needs of rural communities. The healthcare system in rural areas involves a complex interplay between local initiatives and services and broader national structures, with both opportunities and challenges in terms of resources, access, and adaptation to local needs [8]. In rural areas, recruiting and retaining qualified healthcare personnel is challenging [6], and there are long distances to hospitals and other specialist healthcare services. Additionally, in rural areas, where the population is often smaller and healthcare services are more closely connected to the local community, the relationship between patients and healthcare professionals becomes more personal [8, 9]. In this interplay, local healthcare personnel play a crucial role in both delivering direct patient care and redesigning health services to meet changing demands. These complex tasks often occur simultaneously and involve the same healthcare personnel.

This paper offers ethnographic insight into how an existing service, an outpatient primary care emergency clinic, was redesigned to include a Municipal Inpatient Acute Care Service (MipAC) in a rural area in northern Norway and explores the nurse's role in this (redesign) work. MipAC has been politically initiated, and since 2016, all municipalities have been required to offer this service. The MipAC is an alternative to hospitalisation and provides short-term acute medical treatment and care within primary healthcare for stable patients with confirmed diagnoses [10, 11]. The MipAC guidelines allow for significant flexibility in organising the MipAC locally, leading to considerable variation in its structure (i.e., size, number of beds, and medical services provided) and integration within local healthcare services. The competence requirements and work descriptions for healthcare personnel working with MipACs are vaguely described in the national policy guidelines. However, regardless of how a municipality organises the MipAC, a medical primary care physician (PCP) has medical responsibility for the patients enrolled, and nurses’ roles are described in terms of having a 24/7 presence and performing assessments immediately after admission [10].

Previous research on the MipAC has focused on PCPs’ perspectives and has highlighted how PCPs are concerned with the lack of physician presence in the MipAC, especially during evenings and nights when only nurses are present [12–15]. Nystrøm [12] described how PCPs, despite acknowledging nurses' skills, fear that nurses do not have the necessary medical expertise to detect and manage sudden deterioration in inpatient conditions. Studies also show how this reliance on nurses during critical hours leads to hesitation and a lack of confidence in referring patients to the MipAC as a safe alternative to hospital care [12, 13, 15]. Research on nurses' perspectives indicates that they, to a certain extent, share the PCPs' competence concerns about MipAC. Vatnøy [16] highlighted a lack of advanced nursing competence in the MipAC and in municipalities in general. These studies suggest that advanced nursing competence is crucial in caring for MipAC patients [15, 17].

In a previous research paper, based on the same material, we focused on how nurses in an emergency clinic actively contributed to building nursing competence and care arrangements adapted to the national guidelines for MipAC while supporting PCPs’ work in the emergency care clinic [18, 19]. This paper explores how the rural context influences nurses' roles in the clinic and the redesign process.

## Methods

This article builds on empirical material from a comprehensive qualitative study of nurses’ contributions and role in redesigning an outpatient clinic to integrate a generic MipAC model in a rural municipality in northern Norway. The methodological approach is more fully explicated in previously published articles [18, 19]. We were inspired by an organisational ethnographic approach to enhance our understanding of the redesign process [20]. Organisational ethnography, particularly as applied in healthcare research, offers the flexibility to capture how institutional contexts, processes, and factors interact and compete during organisational change [19, 20]. This approach allowed us to examine the dynamics and complexity of healthcare practices during the redesign process, highlighting how social, organisational, contextual and cultural factors shape healthcare work.

The first author was a novice researcher both in the field and in their career and entered the field as an *outsider* [21]. Since the first author was not from a rural area in the north, she was not familiar with the harsh weather conditions and the landscape where the emergency clinic was situated. During the fieldwork, it became evident that the geographical location was not merely a neutral backdrop. Instead, it shaped and influenced the redesign work and the social dynamics within the emergency clinic. These experiences influenced the aim of this paper.

### Study context

One of Norway's most comprehensive health reforms in the last decade was the Coordination Reform, which was implemented in 2012 [11]. The reform aimed to provide better and more effective healthcare by transferring responsibility for more service delivery to primary care services in the municipalities. The reform sought to deliver specialised, high-quality services closer to people’s homes and has contributed to decentralising care and paved the way for several primary and public healthcare reforms to follow ([22];128).

The decentralization of healthcare responsibility has significantly increased the burden on local health personnel, especially in rural areas, requiring them to manage a broader range of medical services, develop new services, and provide care for increasingly ill patients. In Norway, there is both broad political consensus and an expectation from the population that everyone should have equal access to high-quality healthcare services, regardless of where they live [22]. 32% of the population in Norway lives in less central areas with low population density, and approximately half of those live in sparsely populated and rural areas.^1^ The primary health service is historically a generalist service with few specialized services. Maintaining and improving generalist competencies while simultaneously building more specialised competence takes time and is resource-demanding. In addition, there are challenges in recruiting and retaining qualified healthcare personnel in rural areas [6].

The Coordination reform introduced the MipAC, which was a service designed to relieve hospitals by increasing the capacity for monitoring and treating acutely and critically ill but stable patients with confirmed diagnoses in municipalities [10]. In the rural area where we conducted the fieldwork, the MipAC was located in an existing 24-h primary care emergency clinic that served four municipalities, with the nearest hospital located two hours away.^2^ The emergency clinic originally consisted of two examination rooms, a clinical laboratory, and a communication centre. It was redesigned to include the MipAC by adding a new inpatient room with two beds. Severe winter conditions could sometimes create challenging driving situations, and road access was limited, as well as the accessibility of helicopters. During the fieldwork, the first author also experienced that the Norwegian Crucial Communication network^3^ (Nødnett) was unavailable due to faults and that the ambulance service experienced challenging driving conditions during a heavy winter storm. The intermunicipal emergency clinic operated with a team of 14 nurses who worked on a rotating schedule. Each shift had two nurses, PCPs on call outside office hours, and one PCP responsible for MipAC patients during office hours.^4^ All the nurses had their working post at the clinic and lived within the local community. The PCPs’ work was organised differently from that of the nurses. The PCPs worked primarily at local, shared primary care offices. Their day-to-day work was organised around several consultations with their listed patients during office hours, and in addition, they were obligated to take on-call outside office hours shifts at the clinic every 18^th^ day.^5^

Through a national grant scheme, the emergency clinic received funding to redesign the clinic two years before the MipAC became mandatory, and the fieldwork was undertaken during the first year of this grant period. Some of the funds were used to increase the number of nursing staff, and each shift consisted of two nurses on duty, whereas previously, there was often only one. There was also a PCP designated to follow up with the patients during office hours if needed and a PCP on call available outside office hours. The nurses were responsible for monitoring the patients admitted to the MipAC and operating the emergency clinic, including the communication centre and follow-up at the outpatient clinic. The nurses were also involved in completing the initial redesigning processes and switched between these activities and ordinary day-to-day duties as emergency care nurses, which now also included care for patients admitted to the two MipAC beds. The PCPs were integral collaborators alongside the nurses within the clinic, as they were responsible for the consultations and had the overall medical responsibility for the patients.

### Data collection

The fieldwork was carried out by the first author periodically from September 2014 to June 2015. Shadowing [23] was used throughout the fieldwork, and HKH shadowed nurses and PCPs on shifts. Nurses were shadowed more frequently because of their 24/7 presence. The process involved in situ interviews with nurses, nurse leaders, and PCPs in the emergency clinic. HKH observed their tasks, interactions with different materials, such as guidelines, written texts, and people, and where their tasks were performed. During the fieldwork, there was a continuous dialogue with the participants where observations and interpretations were discussed and commented upon. Some of the observations and interpretations were also presented in in-situ and in-depth interviews, where the participants were given the opportunity to comment further. This approach allowed us to access invisible aspects of nurses’ work that are difficult to articulate and are often referred to as trivial and mundane [24–26]. During the fieldwork, the nurses and PCPs were often asked to explain further and elaborate on their actions after specific situations. This helped improve our understanding of their practices and daily work and corrected any misinterpretations. Using shadowing allowed us to discover and capture patterns in the messy, dynamic, and complex context of healthcare work and to examine not only *what* was happening and *how* but also *why * [20, 21, 26]. In addition, the first author and the coauthors (AUO and BN) had frequent conversations during the fieldwork, and the different materials, observations, and interpretations were discussed.

The data material consists of approximately 250 h of observation. Throughout the fieldwork, HKH wrote a substantial amount of field notes. During the observations, she noted quotes and keywords and continually took “writing breaks” to write out more detailed descriptions, which mainly consisted of general descriptions of specific observations or retellings of conversations. Further, it consisted of HKH’s interpretations of these observations and immediate thoughts, and finally, possible follow-up questions and associations [27]. In addition, HKH had numerous in situ interviews with nurses and primary care physicians at the emergency clinic, in-depth interviews with two nurse leaders, and individual in-depth interviews with six nurses. Due to our methodological approach, the interview guide was a dynamic document developed throughout the fieldwork. It was adapted to each interview and served as a ‘checklist’ to ensure that key themes were addressed. Recurring themes included expectations for MipAC, participants’ experiences so far, local adaptations, and their involvement in redesigning the service. Examples of questions ranged from more open-ended inquiries, such as *can you describe what a typical workday looks like for you?* and *what specific new tasks has the implementation of the MipAC model introduced for you?* to more focused questions prompted by concrete observations, such as *I noticed that you used a different form or mentioned making some local adaptations to the national guidelines. Could you elaborate on this and provide examples from specific situations?*

Each in-depth interview lasted approximately 60 min and was conducted during the participants’ work hours. The interviews were audio-recorded and transcribed verbatim. The interview guides were explicitly developed for this study, and two papers based on the same study have been previously published [18, 19].

### Analysis

Data analysis began as the fieldwork started, throughout the fieldwork, and continued into processing data after the fieldwork was concluded. After completing the fieldwork, HKH read all field notes and transcriptions. The initial goal was to identify patterns and actions that illustrated the nurses’ roles in their redesigning efforts and better understand the nurses’ practice within the rural context. NVivo software was used during this stage to organize the extensive data and create broad empirical categories. This analytical process led to a coding framework of descriptive codes drawn from the extensive ethnographical material. This framework was then further refined and discussed collaboratively with the co-authors.

In the early stages, various codes were linked to the geographical location of the emergency clinic. During the initial analysis and fieldwork, we noticed that this context and the rural aspects were not neutral; they influenced aspects such as the working relationships between nurses and primary care physicians; care arrangements; and the competence, role, and position of the nurses in the local community. Our goal in working with this article was to explore these aspects of the material further.

To explore nurses’ role in redesigning the emergency clinic, we were inspired by *the theory of social organisation of healthcare work.* Here, scholars emphasise that high-quality healthcare results from the combined effort of various specialists and specialities, not from individual clinical brilliance alone [24, 25]. Healthcare work is distributed across time and space, and professionals have fragmented understandings of patients, contributing to care through their specific professional knowledge and expertise. Sound patient care requires the right information, competencies, and material resources to be available at the right time and place. According to Allen [24, 28], nurses are central in coordinating distributed activities and making sense of complex and dynamic situations. Allen highlights how nurses draw on their professional expertise, combining formal knowledge and practical experience, to make sense of complex situations and coordinate patient care [28]. This sensemaking process extends beyond individual cognition, involving collective efforts within the organisational context to ensure responsive and well-coordinated healthcare delivery.

In our analysis, we utilise the insights embodied in the social organisation of healthcare work to describe and analyse nurses’ efforts to manage the flow of patients coming into the emergency clinic and adapt their own practices to the MipAC guidelines. Sensemaking plays a crucial role in this context, as nurses interpret and translate the requirements of the MipAC service into practice. When redesigning their service, the nurses engaged in interpretive work to develop routines and workflows that prepared them for new tasks and responsibilities as emergency care nurses. This sensemaking process enabled them to align their practices with the MipAC guidelines while ensuring that the flow of patients and resources met the demands of the new organisational structure. In the redesigned service, they will have extended responsibility in the emergency clinic: to manage/triage patients needing acute care and monitor patients admitted to the new 24/7 service.

However, we argue that the *theory of social organization of health care work* takes the presence of the PCP in the clinic for granted. In our fieldwork, we observed how rural areas played a significant role and that the discontinuity of medical resources impacted the work of nurses, their roles, and the social dynamics within the emergency clinic. To explore these dynamics, we included the concepts of ‘locals’ and ‘cosmopolitans’ [29]. Previous studies have demonstrated that these two concepts are well-suited for describing ideal–typical roles in healthcare and how health professionals navigate them [30]. These roles are not mutually exclusive, and professionals can navigate between them based on context and need. The ‘cosmopolitans’ refer to professionals prioritizing their specialized skills and patient care over the organization. In our interpretation, this description aligns with the role of PCPs in our study, who often appeared more disconnected from emergency clinic due to their episodic presence. In contrast, the 'locals' are more loyal to their organization, less focused on their own profession, and see themselves as part of the local community [29–31]. In our interpretation, the description of the locals aligns with the nurses, who demonstrated a strong commitment to the clinic and the local community and a collective mindset toward building and enhancing competence within the emergency clinic in what they considered essential to strengthening the clinic’s overall expertise.

In line with our ethnographic framework, we followed an abductive approach in analysing the data for this paper, where the theoretical concepts and perspectives guided the coding and further categorisation of the coded data. We especially paid attention to clues and patterns that identified or described rural dimensions, how the nurses and PCPs described and reflected on them, or observations on how they affected the social organisation of their work. We alternated between the empirical data and the theoretical concepts throughout the analysis process until we recognised a pattern in the data. The initial coded text was divided into meaning units, condensed, and abstracted into codes [29]. The codes are abstracts of empirical phrases, quotes, and observations from the fieldwork representative of the pattern in the data.

The codes were grouped into subcategories that offered nuanced interpretations, such as the efforts of nurses to develop the capability to handle critical situations in the absence of PCPs. These subcategories were further aggregated into broader categories like “restricted medical resources” and “continuous nurses’ resources,” which provided a structured understanding of the data. Finally, these categories were synthesized into the overarching theme, the “Rural Dimensions”. Subthemes like “Absent present PCPs” and “The local nurse” illuminated specific aspects of these dynamics, such as the discontinuity of medical resources and the nurses’ efforts to make sense of complex situations and ensure continuity of care. This cohesive analytical process ensured that the findings were deeply rooted in empirical data while reflecting broader theoretical insights into rural healthcare work. (see Table 1 for example).

**Table 1:** Examples of analysis of the interviews

Theme	Rural dimensions					
Subtheme	**Absent present PCPs**		**Learning without doing**		**The local nurse**	
**Category**	Restricted medical resources	Continuous nurses’ resources	Compensating for the lack of learning opportunities and creating safety net		Loyalty and trust	Place/Local community
**Subcategory**	Ability to handle critical situations in the absence of PCPs	The nurses organise the PCP's work	Low bed occupancy, less people and sceptical PCPs	Insufficient practice	I am a rural nurse	Oriented towards the local community
**Codes (meaning unit)**	“Who’s on-call? I’m more alert when there’s an inexperienced primary care physician on duty, especially during poor forecasts. If MipAC patients deteriorate, I need assurance of access to a qualified primary care physician.”	“The nurses know where everything is(…) They are the brains of the emergency clinic, not us, primary care physicians than comes in every third week (…)”	“I question whether some primary care physicians fully understand the service. We are adept at handling a wide range of situations but need the primary care physicians to admit more patients to MipAC for additional practice”	“The criteria for admitting patients are very strict. I seldom encounter patients who qualify. Additionally, there are long distances, and for some patients, it feels safer to send them to the hospital”	“I’m very proud of my workplace. We take great pride in our work. We deliver good services to our residents. The MipAC needs to be a good alternative to the hospital”	“At the end of the day, I need to be able to look people I meet at the grocery store in the eye, knowing I have done what I could. I did my best”

## Results

In the results section, we present three aspects of the rural dimension that are prominent for understanding nurses’ role in the redesign process at the emergency clinic.

### Absent present PCPs

The first aspect of the rural dimension we highlight is how the nurses’ represented continuity in the emergency clinic where access to PCPs is restricted. Being new to the field, the first author expected to observe PCPs and nurses. However, the first author mainly observed nurses at the emergency clinic, and early on, it became clear that the nurses were the continuity of the service. The nurses delivered direct care, managed and organised the day-to-day activities and supported the PCPs’ work. In contrast, the role of the PCP was more episodic, and the PCPs were often inexperienced. Still, the PCPs have a medical responsibility for the patients, and naturally, their decisions and assessments influence the nurse’s work. Our impression was that this *absent presence* of PCPs influenced the working relationship between the nurses and the PCPs, the nurses' role and the redesign of the emergency clinic.

The nurses all lived within the local community. Most of them had their primary position at the emergency clinic and were experienced nurses who had worked there for several years. However, the PCP's role in the emergency clinic was more peripheral because the PCPs primarily worked as local GPs and were required to undertake out-of-hour shifts at the emergency clinic every 18^th^ day. Therefore, the PCPs on duty varied throughout the week. The area had difficulties recruiting and retaining PCPs. Therefore, many PCPs taking shifts at the clinic were interns or temps. The nurses were highly familiar with the facility of the clinic and its routines, and they also knew the local community. In contrast, interns frequently lack familiarity with emergency clinic protocols and local knowledge, while temporary staff are often experienced in emergency care work but unfamiliar with the local community. Then, there were local PCPs who had local knowledge but were more unfamiliar with the facilities at the emergency care clinic. Therefore, nurses play a vital role in the daily operations of emergency clinics, particularly in assisting and supporting novice and experienced PCPs. One local PCP said:

*Excerpt 1*

*It is enormous support to have the nurses on the team (…) They know where everything is, equipment, forms, all that. They even know where people live and the medical history of some of the patients(...)” (*in situ *interview, physician).*

From the beginning of the fieldwork, it became apparent that the nurses played a crucial role in maintaining the smooth functioning and coordination of the emergency clinic, whereas the PCPs focused on delivering consultations and treatments. We argue that the nurses took on a great responsibility in ensuring that the service was well organised. However, being in a rural context meant that access to experienced PCPs was limited, the distance to hospitals was significant, and access to other emergency care services, such as ambulances and helicopters, was often unpredictable. Consequently, the nurses’ assessment of the medical resources available was a crucial part of their sensemaking process, enabling them to evaluate the clinic’s capacity to manage patients effectively and prioritize actions accordingly.

*Excerpt 2*

*“A large portion of our work is in the emergency call centre. Here, we use our clinical judgment to make several assessments in just a few seconds. We promptly make an image of the situation and the patient. We consider calling the PCP or sending the patient directly to the hospital. To send a patient directly to the hospital involves contacting the ambulance and tracking it locus in the region. [...] Furthermore, we have to find out who (PCP) is on duty; if they are experienced, we may ask them to come to the emergency clinic, but if they are new, we may hesitate […]. I feel like we are pretty good at this. Most PCPs trust us; when we call the emergency unit at the hospital, they know it is serious [...] . I often feel like we have to coach the PCPs(...)" (Interview, Nurse 6)*

This quotation illustrates an essential contextual element in rural nursing: how nurses organise and coordinate what they consider appropriate resources for each emergency call and how they include knowledge of the rural context. Drawing on our theoretical concepts of the social organisation of healthcare work, which emphasise the organising and coordinating roles of nurses in the provision of care, it becomes evident that nurses not only manage their own tasks but also ensure that the PCPs’ work can be carried out effectively despite their presence in the clinic being episodic.

Excerpt 3

*I like to be “on top of things.” For example, if I’m having the weekend shift, I check who I’m working with. Are we several experienced nurses, what PCPs call, and so on. In addition, in winter, especially. How is the forecast? Are there storms coming? (…) If a helicopter can’t land, ambulances are on other missions, can’t get there, or even worse, are parked. I feel better knowing that we are more qualified health care personnel on shifts. (*In situ* interview, nurse)*

Nurses gather and assess information they consider relevant to managing incoming patient calls. The excerpt above illustrates how nurses engage in a sensemaking process incorporating rural contextual factors into their work organising and coordinating. Drawing on their familiarity with the local community and the emergency clinic, the nurses quickly construct images of patients’ conditions, informed by firsthand experience managing critical situations with limited medical resources, long distances to hospitals, and harsh weather conditions.

Our analysis highlights how this sensemaking is central to the redesign process, as the nurses integrate their knowledge and experience of these rural challenges into planning and implementing the new MipAC service. A key aspect of their sensemaking involves a “worst-case scenario” mindset—anticipating how patient trajectories could unfold, especially in cases where a patient’s condition might deteriorate rapidly while the PCP is absent or inexperienced. By envisioning these potential scenarios, the nurses identify what resources, routines, and contingency plans must be in place for them to manage the reorganised service in a way they consider “sound medical care”.

### Learning without doing

The second dimension of the rural aspect we want to highlight is the lack of practice and having to learn without doing. Learning by doing (on-the-job training) assumes that people learn from ongoing experiences, perform actions in everyday work, observe, ask questions, and learn from what they do. Being in a rural area naturally means a smaller population and, consequently, fewer potential patients. This potentially limited patient volume reduces opportunities for hands-on experience and on-the-job training for new services.

The MipAC was new during the fieldwork and had a low bed occupancy. Although the emergency clinic nurses appeared to be experienced emergency care nurses, caring for the MipAC patients potentially required different types of attention and skills. Before the MipAC was introduced, a typical MipAC patient was initially examined at an emergency clinic and referred to hospitals for treatment. During the first shadowing period, some nurses expressed concerns about not having enough practice and experience caring for MipAC patients over time. Several nurses expressed concerns about being responsible for the MipAC patients in a way they considered “sound medical care” and were eager to practice more to freshen up their skills.

*Excerpt 4*

*She enters the empty MipAC room (…) ”I want to do my job well, but it is been many years since I worked in a hospital or nursing home. I feel more well trained for a car accident and full chaos, and I need to feel that I’m just as-prepared for this MipAC patient. I need to get MipAC “down to a fine art”, but there are too few patients.” We are left standing, looking at the two empty made-up beds. (Fieldnote, observation, nurse and HKH)*

Our impression was that the nurses anticipated that they could find themselves in situations where they had to manage the challenge of simultaneity—balancing 24/7 observation and care of MipAC patients while also operating the emergency call centre and assisting doctors with patients in acute need of help at the emergency. This was further exacerbated by what is described under the concept of the *Absent presence of PCPs*, namely the lack of consistent access to experienced doctors. The nurses gave the impression that they were skilled at running the service efficiently despite limited medical resources and were capable of handling complex emergencies quickly and safely. At the same time, they expressed that their main concern was gaining as much practical experience as possible to provide sound and reliable care for the patients admitted to the MipAC at the same time. The quotation below illustrates this concern among the nurses. Their knowledge about the rural context and unstable access to experienced PCPs were embedded in their concerns:

*Excerpt 5*

“*We are well trained for a variety of situations, and many of us nurses have experiences and knowledge that we have built up over several years. We do our best for our patients every day. That is why we’re here. Not to provide MipAC or to deliver a service. No, we’re here for the patient. Do not forget that. I feel that I have to do my best every single day. I think we nurses in the periphery assess things differently than those in cities do. For example, we do not have the PCPs here all the time, these specialists, etc., and we have to be able to handle the situations. This will also be the case with MipAC; I just feel that we need to gain more experience and get more practice” (Interview, nurse).*

The nurses were concerned with improving their competence in emergency care by “learning by doing”. However, this was challenging, as few patients were admitted to the MipAC. During the initial phase of operating MipAC, neither the PCPs nor the nurses got much practice in caring for MipAC patients.

Lacking practical experience in caring for unstable patients in danger of deterioration and facing an inconsistent presence of experienced PCPs, the nurses prioritized the development of emergency care nursing procedures and establishing collaboration plans with prehospital emergency services in their redesign efforts. In these efforts, the nurses drew on rural contextual factors, past experiences, and professional competence to reconfigure their work processes and adapt to the national MipAC guidelines, tailoring them to fit the realities of their emergency care clinic. A key focus was creating a framework that compensated for gaps in practical experience and ensured the clinic could function effectively under challenging circumstances. The nurses established a safety net by developing comprehensive plans, procedures, and routines to support seamless operations and collaboration. Our impression was that these adjustments were also to reinforce their confidence and reassure the PCPs.

### The local nurses

The third aspect we highlight is *the local nurses*. In our study, we recognised that a place is not a neutral backdrop. In this case, the place is characterised by close-knit communities, long distances to hospitals and other health care services, harsh winter conditions, the possibility of closed roads, an indigenous population, mountainous terrain, military presence, soldiers, nature, and recreational activities such as fishing, hunting, and hiking. Based on our data, our understanding was that the nurses, to a greater extent than the PCPs, were oriented towards the local community.

Most of the nurses had been actively involved in different service redesigns over the last few years. They took great pride in their service and expressed ownership of the emergency clinic and the new MipAC. According to the nurses, they had been central in developing a new and more comprehensive emergency service than the previous one that they considered could provide “sound medical care” and was capable of handling demanding acute situations with limited emergency care resources available. Frequently during fieldwork, statements such as this quote were commonly heard.

*Excerpt 6*

We built this service. So of course, we are proud of it. (...) We provide good services to our residents (...), and I feel that they trust us” (Interview, nurse).

This quote also highlights an important aspect of the context of the redesigned emergency clinic. Based on our observations, the nurses had a strong sense of affiliation with and ownership of the emergency clinic. They were deeply committed to ensuring that the clinic was perceived as a provider of high-quality medical services within the local community.

The service and its overall expertise have evolved through several years of continuous development and adaptation. When explaining this to the first author (HKH), the nurses expressed their concern that residents might lose confidence in them if the redesigned emergency clinic failed to maintain the same standard as the previous emergency services. According to the nurses, the service continued to be trusted by “their residents,” the nurses were determined to preserve and uphold this trust as they worked to integrate the MipAC into the clinic. As “locals,” their loyalty was rooted in providing excellent services to those living in rural areas.

Excerpt 7

“(...) no one taught us about “the small things”. Running an emergency clinic. We had to figure that out for ourselves. (...) However, I think many of us have more ownership of the emergency clinic here than most people have to their workplace. (…) when I say we, I mean us nurses who work here. Because as I have said many times. If not us, then who? No one, I will tell you (....) So, this emergency clinic, is our creation.” (Interview, nurse)

As a group, the nurses appeared to have a collective mindset, frequently using phrases such as ‘we nurse,’ referring to ‘our emergency clinic’ and ‘our residents.’ In contrast, the PCPs appeared, more like external contributors. This could be attributed to the peripheral role of the PCP in the emergency clinic, which was reproduced by including the MipAC in this service. The PCPs had a more episodic presence; it could be weeks between each shift they had at the clinic, and the work at the clinic was also limited to brief consultations. Apart from having medical responsibility for the patients, the PCPs were generally not involved in the daily running of the emergency clinic and, thus, were less involved in the MipAC. When the PCPs talked about the MipAC, it was often more limited to medical questions and the responsibility that the beds entailed for them. Several PCPs expressed frustration over how they felt the municipality imposed the MipAC on them.

Excerpt 8

“What this organisation means in practice is that, when we are on emergency clinic duty, we are not to follow up on the MipAC patient unless the patient becomes critically ill or the nurse assesses it to be critical. In my everyday work, I don’t relate much to it yet. I know that it is now an offer. (In situ interview, physician)

Several nurses expressed their anticipation of greater PCP involvement in redesigning the emergency clinic and hoped that the PCPs would take more ownership. As the excerpt illustrates, the PCPs had more limited responsibility for daily operations and running the emergency clinic and, to a lesser extent, took ownership of the emergency clinic compared to the nurses. The PCPs’ absence and peripheral role explain their lack of involvement in the redesigning process. In contrast, the nurses who represented continuity in the clinic and were “locals” were more committed to ensuring that the redesigned emergency clinic met the needs of “their residents”.

## Discussion

Our results highlight how rurality shapes the redesign of an emergency clinic into a MipAC model, emphasizing three key dimensions. *First*, Nurses provide continuity in the clinic, while PCPs’ presence is episodic due to the local shift arrangement. In addition, because of challenges in recruitment and retention, many PCPs on duty were interns or temps, often inexperienced and unfamiliar with the rural context. In response, nurses leverage their extensive local knowledge, assess available medical resources, prioritize actions, and guide PCPs to ensure adequate care, compensating for the lack of consistent medical presence. *Second*, The low patient volume typical of rural areas limits opportunities for nurses to practice and develop new skills, in this case caring for MipAC patients 24/7. Our impression was that this created concerns about the nurse’s ability to handle complex cases, especially alongside acute emergencies. Nurses develop detailed procedures and collaborative plans to mitigate these challenges, ensuring patient safety and compensating for their limited hands-on experience. *Third*, the nurses also demonstrate a strong connection to their community, taking pride in their work and feeling responsible for maintaining public trust and delivering high-quality care in the local community.

The *firs*t prominent rural dimension is restricted access to medical resources, which contrasts with the continuity of nursing resources. Although PCPs hold formal medical responsibility for patients in both emergency clinic and MipAC [30, 31], our data—consistent with prior research—demonstrate the absence of PCPs, especially during the evening and night [12–16]. We argue that this “absent presence” of PCPs, where they bear substantial medical responsibility as mandated by governmental guidelines but have a limited physical presence, creates a paradox. This paradox requires nurses to navigate the complexity of delivering emergency care with minimal direct PCP involvement while simultaneously ensuring continuity and coherence in the emergency clinic and adapting these services to the MipAC. This paradox is further amplified in the Norwegian context, where there is a strong societal expectation that rural health services should maintain high quality and provide accessible medical care regardless of geographic location [22].

Allen [24] emphasises the crucial role of nurses in assembling and aligning competencies, appropriate information, and materials to meet patients’ needs and manage patient trajectories This organising and coordinating work is complex in itself and becomes even more complex when new activities and practices are introduced in the existing service [18, 19]. Furthermore, the episodic presence of PCPs introduces a potential weakness in the organisation of care, making nurses’ coordination efforts and continuity even more critical. As previously demonstrated [19], rural nursing involves the ability to anticipate and prepare for potential crisis situations by anticipating patient trajectories, creating safety nets, preparing for potential emergencies, and ensuring that the necessary resources are available. In this article, we elaborate on these findings, showing that the nurses in this study make assessments based on their evaluation of available medical resources and their sensemaking processes to anticipate potential worst-case scenarios. Our findings further indicate that nurses actively engage in sensemaking to manage the complexity of their roles, ensuring that necessary resources and information are available for the *absent present* PCPs. We argue that this “proactive” approach is essential to maintaining high-quality care and draws attention to the significant role of nurses in the redesign of the clinic.

The *second* prominent dimension was a lack of practice in caring for MipAC patients. The low bed occupancy in MipAC was due to a combination of factors. Being in a rural area naturally means fewer potential patients, and finding suitable patients who meet the admission criteria was challenging. Previous research has shown that this often led to extensive discussions regarding patient eligibility [12, 19]. As a result, not enough patients were admitted, leaving nurses with insufficient practice in handling the variety of patients who could qualify for MipAC. This lack of hands-on experience is especially critical when a new service is introduced [30].

Earlier research has suggested that nurses do not have the necessary medical expertise in managing sudden patient deterioration in MipAC [11–15] and lack advanced nursing competence [16]. Our study presents a compelling issue in this regard; our results illustrate how nurses actively work to be able to handle advanced emergency care in the rural MipAC. Due to the limited opportunities to practice and build experience, nurses developed safety nets to compensate for the lack of learning opportunities. These safety nets were designed to ensure that the redesigned service could provide care in a manner they considered medically sound in light of the organisational resources they had available [19]. Through sensemaking, nurses interpreted the complexities of their roles and the organisational context, enabling them to develop strategies to navigate these challenges. The nurses worked hard to build their skills, competence, and confidence in caring for these patients. To do so, they draw on their clinical and organisational knowledge, take into account contextual factors, and prevent worst-case scenarios by establishing extensive safety networks such as procedures and routines to prevent potential issues [19]. Building clinical competence to support their work in caring for MipAC patients and, at the same time, support the PCPs’ work. Even though the nurses, to some extent, shared the PCP’s competence concerns, they took proactive measures and assumed extensive responsibility to reassure both themselves and the PCPs that they could support the PCPs’ work and manage potential deterioration in the patients’ condition.

The *third* dimension in the redesign process was the nurses’ strong orientation toward the municipality and their eagerness to adapt the service to meet local needs based on their interpretation of those needs. The episodic presence of PCPs in the clinic contributed to a perceived lack of commitment and engagement in this process. As the MipAC was situated within the emergency clinic, the PCPs’ peripheral roles limited their involvement in both the redesign and the operation of the new service. This dynamic left nurses to take on a central role in shaping and implementing the redesigned MipAC.

Nurses in rural areas face challenges distinct from those in more centralised healthcare settings, where physician resources and access to services are more consistent [9]. Our study indicates that the nurses expressed a deep understanding of the local community and healthcare environment, which they perceived as surpassing that of the primary care physicians. This sense of responsibility and ownership of the clinic, shaped by their interpretation of the community’s needs, appeared to play an important role in how they approached the redesign process. The nurses communicated that their efforts were aimed at ensuring the service aligned with local expectations and demographics, place and the local organization of health services.

By applying the concepts of “locals” and “cosmopolitans,” we argue that the governmental MipAC guidelines primarily address the concerns of “cosmopolitans” rather than “locals.” In our interpretation of these concepts, cosmopolitans are typically oriented towards broader health care systems and medical standards, whereas locals are more focused on the local adaptation of governmental initiatives to meet specific local needs [31, 32]. The MipAC guidelines emphasise clinical criteria, medical professional guidelines, assessments, and the physician’s role in maintaining medical responsibility for enrolled patients and the need for a 24/nursing presence. However, they allow significant flexibility in how services should be organised locally, offering little guidance on how this process should be carried out, who is responsible for it, or the specific role of healthcare personnel in contributing to it. While this flexibility can be seen as advantageous, particularly in allowing adaptations to local contexts [33, 34], a key question in rural healthcare is whether this vagueness ultimately supports or undermines local redesign processes.

Our study indicates that in rural areas with limited access to medical resources, expecting PCPs to participate in reorganizing services and establishing new initiatives like MipAC can be quite challenging, spite their medical responsibilities. Our results indicate that rural nurses made significant efforts to redesign the service within their everyday work. While the flexibility of the guidelines allowed for local adaptations, we believe it also put pressure on nurses to bridge the gap between national standards and local realities. Further research is needed to explore how these dynamics unfold in similar settings, particularly regarding reliance on nurses to address these discrepancies. A key finding in our study is that nurses relied heavily on their organizing work to facilitate these adaptations; however, this competence often goes unrecognized. We argue that acknowledging and valuing this organizational work is essential for enhancing the sustainability and effectiveness of such services in rural contexts.

Several studies support the notion that redesigning healthcare services requires a thorough understanding of the context in which they operate [31, 33]. Our study shows that nurses, as “locals,” are uniquely positioned to facilitate these redesign efforts because of their familiarity with and knowledge of both the clinical environment and the clear interpretation of the community’s needs. In their redesign efforts, they are vital for reshaping broader health care and guidelines to meet the premiss and needs of rural communities.

We argue that a thorough understanding of local conditions is essential for redesigning existing services to meet changing needs. This requires not only a deep understanding of one’s own work and role but also insight into the work of others, alongside extensive experience and knowledge of the overall service and local context — something our data show nurses possess. Additionally, we emphasise the importance of recognising that the rural context into which MipAC is introduced is not neutral, even though the guidelines may suggest otherwise. This awareness is crucial for those involved in redesigning services, municipal authorities, and the development of health policies.

### Possible implications for practice and further research

We argue that nurses, through their organizing work and sensemaking, not only managed patient care and ensured smooth and safe patient trajectories but also actively engaged in redesigning the emergency care clinic. By doing so, they adapted the service to meet local needs while aligning it with national requirements, effectively driving the development of the rural service forward. The nurses’ strong dedication and orientation towards the local community explain why they willingly took on extended responsibilities, highlighting their critical role in shaping a service that remains both locally relevant and nationally compliant. Our findings illustrate that the scope of practice for nurses is more extensive in rural areas and should receive greater attention at the national level, particularly in the shaping of health policy. These findings also highlight the need for further research to enhance our understanding of nurses organizing work, and the challenges and opportunities in this field. They may also provide valuable insights for other contexts where similar healthcare dynamics are present.

#### Limitations

We acknowledge that this study focused primarily on the work and contributions of nurses. However, an essential aspect of the context involves the roles of other professionals. Understanding the complexity of the redesign process requires examining how different professions collaborate, as no single profession operates in isolation. We consider it both a strength and potential weakness that we focus on the nurses’ work. We did not have in-depth interviews with PCPs (observational data and in situ interviews only); we have not interviewed municipal leaders, politicians, or other health services on the subject of redesigning the emergency clinic, which could be of interest for further research.

## Data Availability

All participants provided their informed consent to participate. However, the data sets generated and analysed during the current study are not publicly available due to the lack of obtained consent among the participants to share raw data but are available in Norwegian from the corresponding author upon reasonable request. Anonymity and confidentiality were assured according to standard procedures, and the participants’ names and other directly identifying information were anonymized in the written text.

## Abbreviations

MipAC: Municipal inpatient Acute Care service
PCP: Primary Care Physician

## Footnotes

1 Centrality classes are used to classify municipalities according to their function in the national context. Centrality class 4–6 typically indicates fewer central areas with lower population density and less development than areas in higher centrality classes (SSB population, 2023).

2 It could be a 3-h drive in the winter with poor driving conditions.

3 Nødnett connects the Norwegian emergency resources. Nødnett has made it possible to share information, build common situational awareness and collaborate during emergencies: Nodnett.no.

4 There was one dedicated PCP responsible for follow up MipAC patients during daytime, this was a local PCP and was only present during these follow ups.

5 This varied. Some PCPs took shifts more often, some where exempted, and many of the interns and temps volunteered to take several shifts.
